# Plant-Derived Extracellular Vesicles: Current Findings, Challenges, and Future Applications

**DOI:** 10.3390/membranes11060411

**Published:** 2021-05-29

**Authors:** Nader Kameli, Anya Dragojlovic-Kerkache, Paul Savelkoul, Frank R. Stassen

**Affiliations:** 1 Department of Medical Microbiology, School of Nutrition and Translational Research in Metabolism (NUTRIM), Maastricht University Medical Center, 6200MD Maastricht, The Netherlands; n.kameli@maastrichtuniversity.nl (N.K.); anya_dk@hotmail.com (A.D.-K.); paul.savelkoul@mumc.nl (P.S.); 2 Department of Medical Microbiology, Faculty of Applied Medical Sciences, Jazan University, Jazan 45142, Saudi Arabia; 3 Department of Medical Microbiology and Infection Control, VU University Medical Center, 1007MB Amsterdam, The Netherlands

**Keywords:** extracellular vesicles, plant EVs, intestinal homeostasis, immunomodulatory effects, therapeutic applications

## Abstract

In recent years, plant-derived extracellular vesicles (PDEVs) have gained the interest of many experts in fields such as microbiology and immunology, and research in this field has exponentially increased. These nano-sized particles have provided researchers with a number of interesting findings, making their application in human health and disease very promising. Both in vitro and in vivo experiments have shown that PDEVs can exhibit a multitude of effects, suggesting that these vesicles may have many potential future applications, including therapeutics and nano-delivery of compounds. While the preliminary results are promising, there are still some challenges to face, such as a lack of protocol standardization, as well as knowledge gaps that need to be filled. This review aims to discuss various aspects of PDEV knowledge, including their preliminary findings, challenges, and future uses, giving insight into the complexity of conducting research in this field.

## 1. Introduction 

In recent years, extracellular vesicles (EVs) have gained the interest of many scientists and experts. EVs are nano-sized, lipid bilayer-enclosed extracellular structures that are released from a variety of cell types [1,2] and can be characterized by either size, density, biochemical composition, or conditions/cells of origin [3,4]. EVs are not only of interest due to their role in intercellular communication [5], but are also being increasingly appreciated due to their complex biochemical composition [6], their role in health and disease, and their potential future applications, including their use as diagnostics tools and therapeutics [2,7]. Extracellular vesicles have been identified from numerous sources, including bacteria, mammalian cells, and plant cells [8], and have been successfully isolated and characterized using a number of techniques. However, researchers are yet to create a gold standard technique for the isolation of EVs, resulting in increasing amounts of research and hypotheses [9]. Another factor that makes EVs a current hot topic in research is their level of diversity, including size, morphology, biogenesis, and biological functions. There are many unknown or uncertain qualities of EVs, and these qualities are what keep challenging many researchers today, adding to this ever-growing field of research [10].

As previously mentioned, EVs have been identified from a number of sources. For example, they are known to be produced by bacteria, as well as being present in biological fluids from mammals, such as blood, urine, and plasma [9]. EVs are also able to transfer their internal cargo, including proteins, lipids, and nucleic acids, to other cells and exert a biological effect [9,11,12,13,14]. While mammalian-derived EVs have been well documented [1,15], EVs from the plant kingdom are not as well documented or characterized. However, in recent years, the potential roles of plant-derived extracellular vesicles (PDEVs) in relation to health and disease have been investigated more intensely.

The interest in plant-derived extracellular vesicles has increased over the last few years, particularly in relation to the gut microbiome and homeostasis. It is well known that although the composition of the gut microbiota is rather stable throughout the course of an individual’s life, changes can be induced by changing the diet [16,17]. For example, it has been found that plant-based diets can promote more bacterial diversity in the gut [18,19], and this may link to an individual’s immunity and the development of gastrointestinal diseases, such as inflammatory bowel disease (IBD) [19]. Interestingly, recent data suggests that exosome-like vesicles from plant sources may play a major role in maintaining intestinal symbiosis and homeostasis [15].

In addition to their role in regulating intestinal homeostasis, in vitro and in vivo experiments have revealed that PDEVs may exhibit anti-inflammatory effects, anti-cancerous effects, and are known to play an important role in cell–cell communication [15]. More importantly, PDEVs are also gaining interest due to their lack of toxicity to and ease of transport through human cells, providing potential opportunities for drug delivery and therapeutics [20]. This review aims to discuss the importance of plant-derived vesicles and current findings in this field of research. Additionally, the future applications of PDEVs will be discussed and speculated, as well as the current challenges and knowledge gaps in the field, which will give insight into the complexity associated with PDEV research.

### 1.1. Plant Vesicles: Current Findings and Importance

Due to the potential applications of plant-derived vesicles, researchers have already conducted studies, which have had intriguing results both in vitro and in vivo. One of the earlier studies conducted on PDEVs examined the effect of grape EVs on intestinal stem cells [21]. After the verification of grape-derived EVs using common procedures including electron microscopy, size distribution, protein analysis, and lipid analysis, grape EVs were labelled using an infrared fluorescent membrane. After tracking the movement of these grape EVs in mice, results indicated that these EVs are able to migrate through the intestinal barrier. Additionally, grape EVs were taken up by intestinal stem cells, where they stimulated stem cell proliferation and self-renewal, indicating that edible PDEVs may have direct influences on the state of our intestinal health [21]. To further verify this, the same study also found that mice which were administered grape-derived EVs were also protected against dextran sulphate sodium (DSS)-induced colitis, compared to mice which were administered PBS as a negative control. The data indicated that mice which were fed PDEVs once a day had an improved mortality rate, and a reduction in the progression of the DSS-induced colitis. Such findings provide implications for the role of PDEVs in diseases related to the intestinal system.

In a different study, Raimondo and colleagues showed the anti-neoplastic properties of nanovesicles derived from citrus lemon [22]. Various cancer cell lines, including a human lung carcinoma cell line and a chronic myeloid leukemia cell line, internalized these citrus vesicles with a subsequent inhibition of the growth and viability of the tumor cells, suggesting a potential role for these vesicles alongside regular cancer treatment. Similarly, Kim et al. [23] have found that EVs derived from plant sap can exhibit cytotoxic effects on tumor cells. These findings further suggest that PDEVs may have a direct influence on biological pathways related to health and disease, making them an area of interest for researchers.

Not only has it been shown that PDEVs influence intestinal cells and cancer cell lines, but data further suggests that other tissues in the body may be affected by dietary PDEVs. One such finding has shown that ginger-derived nanovesicles can protect against alcohol-induced liver damage [24]. Here, researchers found that mice fed with ginger-derived nanoparticles had reduced lipid droplets in the liver, as well as lower levels of liver triglycerides when compared to mice that were not gavage-fed with ginger vesicles. Intriguingly, after labelling the ginger nanoparticles, fluorescent signals were found only in the liver, indicating that ginger EVs entered the liver, which was further confirmed using immunostaining. These findings interestingly suggest that hepatocytes are a major target of ginger-derived EVs. In contrast, in a different study, the same research team found that grapefruit-derived EVs were primarily taken up by Kupffer cells, suggesting a clear preference for specific organs depending on the source of the PDEV. In addition, findings such as these further indicate that plant-derived vesicles may have a variety of effects on different organs and tissues, as well as homeostatic regulation in the body. This supports the idea that PDEVs may play a role in the connection between our gut microbiome and remote organs and how this may influence the health of other organs within the body.

**Figure 1:**
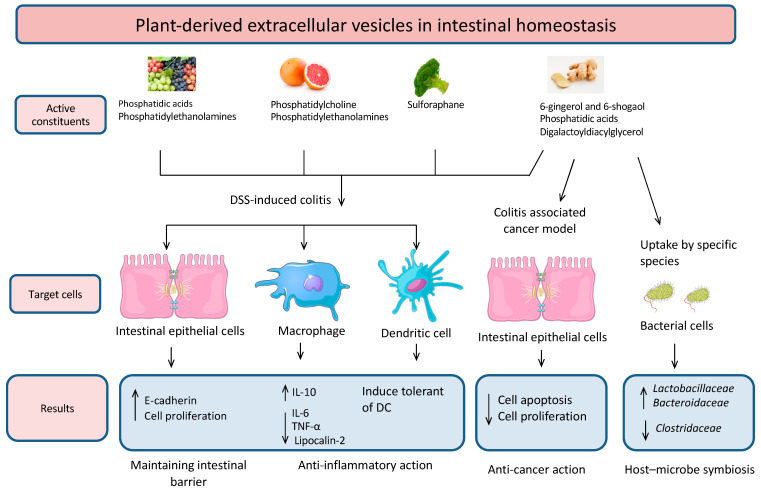
Biological functions of plant-derived EVs (grape, broccoli, ginger, and grapefruit) in intestinal tissue and microbial composition. These EVs have a valid role in protection against inflammation and intestinal permeability, and they also participate in shaping the microbial composition.

Furthermore, it has also been demonstrated that plant-derived vesicles can influence immune cells, suggesting that not only do these PDEVs affect various tissues and organs in the body, but they may have a direct impact on our immune system. For example, it has been shown that PDEVs are taken up by macrophages and exhibit anti-inflammatory effects [25,26]. In one of these studies, colitis was induced in mice using DSS, and the mice were subsequently administered ginger-derived EVs. Results indicated that these PDEVs exhibited anti-inflammatory effects due to an observed decrease in lipocalin-2, which is used as a biomarker for intestinal inflammation [25]. Ginger-derived EVs also increased the expression of E-cadherin, a cell–cell adhesion molecule that plays a crucial role in maintaining the intestinal barrier, which is implicated in intestinal disease [25]. Additionally, it was found that mice treated with these ginger EVs also showed significantly lower levels of pro-inflammatory cytokines, such as TNF-α and IL-6, as well as increased levels of the anti-inflammatory cytokine IL-10. Similar findings have been observed for grapefruit-derived nanovesicles and indicate that plant-derived vesicles may be interconnected with our immune system and the regulation of our health (Figure 1).

Interestingly, research on edible plant-derived vesicles has also found that these EVs may be obtained from various parts of a plant. It has been shown repeatedly that EVs can be successfully isolated from both the juice of plants [22,27,28,29], and the flesh or roots of plants [18,20,24]. Additionally, EVs have been isolated from the seeds of plants [30] and dried plant material [8]. Furthermore, it has been found that extracellular vesicles exhibit high levels of stability in various environmental conditions, and are able to resist degradation from certain digestive enzymes [31]. While the findings regarding PDEV stability varies between studies, some initial findings already provide interesting insights into this characteristic. With further experimentation on PDEV stability and function in various environments, this information may be useful for better understanding the future use of PDEVs in therapeutics.

Finally, there is one other feature of plant-derived vesicles that has been expanding recently: the internal cargo of plant-derived vesicles, including proteins, lipids and RNAs. Of particular interest are the microRNAs (miRNAs) that have been observed in PDEVs. Detailed analyses have shown that plant EVs have a rich and diverse population of miRNAs [18,28,32]. One study that investigated the miRNA composition of plant-derived vesicles from 11 different fruits and vegetables identified 418 different miRNAs among the 11 samples, many of which have a multitude of potential target genes. For example, genetic analysis predicted that some of the PDEV miRNAs may be able to target mammalian genes involved in the regulation of inflammatory factors, including IL-5 and IL-6 [29], suggesting that these plant vesicle-derived miRNAs are able to directly target and potentially regulate mammalian mRNAs and biological pathways. This is supported by another study that also analyzed the effect of ginger EV-derived RNAs and found that these RNAs can bind to mRNAs from bacteria in the gut and influence biological pathways. With these results, it is interesting to consider the potential roles that these plant vesicle-derived miRNAs may play in host health and disease. It is well known that these miRNAs are important in regulating a variety of biological processes in plants, such as growth and stress responses. Therefore, there may be some interesting connections to human diseases [29]. Another interesting example that has linked plant-derived miRNAs to human health is one study that found that miRNA-2911, which is abundant in the honeysuckle plant (*Lonicera caprifolium*), is able to inhibit influenza A viruses by preventing the production of the virus-encoded proteins PB2 and NS1 [33]. Results such as these already suggest many of the potential medicinal and health-promoting effects that plant-derived vesicles and their miRNAs may have once they are better understood.

Considering the promising results that have been obtained from in vivo mouse models and in vitro experiments (Table 1), further research on plant EVs is recommended, as they may provide a number of potential applications that warrant further investigation.

### 1.2. Plant Vesicles: Potential Applications as Drug Delivery Systems

Much of the current research on plant-derived EVs suggests that these vesicles can have direct effects on recipient cells by transfer of their internal components, including lipids, proteins, and RNAs. In addition to this, further investigation has demonstrated that these PDEVs can be used to transfer medicinal agents, making the future use of PDEVs as nanovectors promising [20,26,36]. The creation of specific drug delivery mechanisms is necessary to improve treatment options for patients with a variety of diseases [20,36,37]. Due to their lack of toxicity and ease of transport through mammalian barriers, plant-derived vesicles are being extensively studied as a novel method to achieve this, and findings already suggest that these vesicles may be excellent candidates for nano-delivery [20,26].

**Figure 2:**
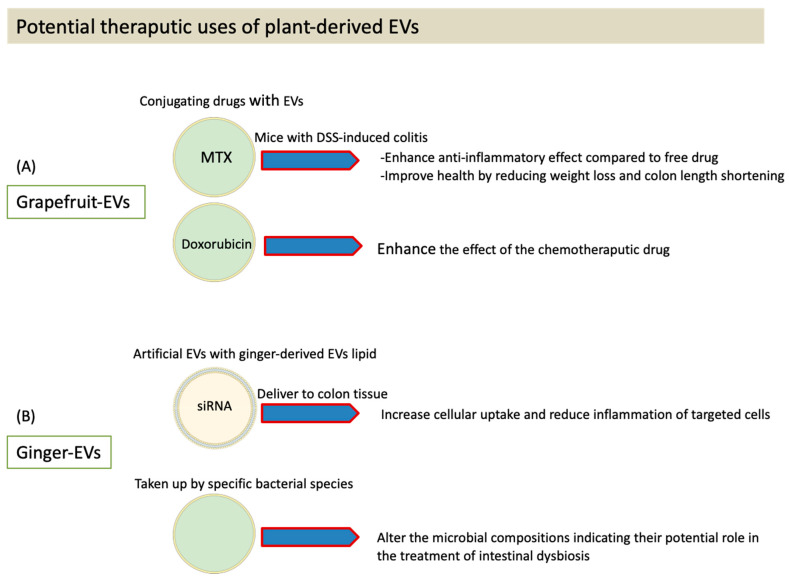
Plant derived EVs (ginger and grapefruit) as drug delivery systems and therapeutic agents. (**A**) Grapefruit. (**B**) Ginger.

One of the plant food sources that has been investigated for its use in the nano-delivery of drugs is grapefruit (Figure 2). When compared to liposomes, grapefruit-derived nanovectors were shown to be more effective at delivering methotrexate (MTX), a common anti-inflammatory drug, and they exhibited more specific targeting of intestinal macrophages in mice [26]. Results also showed an improvement in the health of mice with DSS-induced colitis, as indicated by a reduction in weight loss and colon length shortening [26]. Similarly, results from another study indicate that grapefruit-derived vesicles can deliver DNA and proteins, including antibodies, to target cells without inducing a cytotoxic response. Additionally, it has been demonstrated that chemotherapeutic drugs, such as doxorubicin, can be delivered to targeted tumors using plant-derived EVs, such as EVs from grapefruit, while also enhancing the effects of the drug (Figure 2) [20]. These preliminary findings have created a solid base for the use of PDEVs in nanotechnology.

Interestingly, it has also been found that ginger-derived nanovesicles are able to deliver a specific siRNA to colon tissue, resulting in reduced inflammation of the target cells [38]. It has also been demonstrated that certain PDEVs may be preferentially taken up by one strain of bacteria over another [18]. Future studies are needed to elucidate the mechanisms underlying this cell/bacteria specificity.

### 1.3. PDEVs for the Delivery of Certain Compounds

Evidence indicates that the use of plant-derived EVs for the nano-delivery of drugs offers a variety of benefits, including safety and efficiency. Since these plant-derived vesicles come from natural food sources, they have already demonstrated a lack of toxicity in humans [38]. Additionally, compared to more commonly used nano-delivery methods, PDEVs appear to have fewer complications and allow for more specific targeting of cells than synthetic lipids. This has been demonstrated for a number of plant-derived vesicles, including ginger and others. For example, Teng et al. found that ginger-derived EVs are preferentially taken up by *Lactobacillaceae*, whereas grapefruit EVs are preferentially taken up by *Ruminococcaceae*, which has interesting implications for the potential future use of plant-derived vesicles for the delivery of microbial agents [18]. The same group also found that providing mice with ginger-derived nanoparticles led to increases in the gut microbial population of *Lactobacillaceae* and *Bacteroidaceae*, while the population of *Clostridaceae* decreased (Figure 1). These results suggest that plant-derived vesicles can alter the microbial composition of the gut, indicating their potential use for the treatment of intestinal dysbiosis and associated diseases [18].

Additionally, it has been shown that plant-derived vesicles are not able to pass the placental barrier. This was observed when grapefruit-derived nanoparticles were injected into pregnant mice. Here, it was found that following injection, the grapefruit-derived vesicles did not pass into the placenta or interfere with the pregnancy [20]. These results have exciting implications for the use of plant-derived vesicles as systems for the delivery of drugs in pregnant mothers, although many other plant sources would have to be investigated before conclusions can be drawn from this. Nevertheless, this preliminary data offers additional insights into some other possible benefits of using grapefruit-derived EVs. It is therefore understandable that there is an interest in furthering the knowledge on the use of plant vesicles as drug delivery systems, considering that many other plants may offer the same valuable benefits.

The already-existing body of evidence on the use of PDEVs for nano-delivery offers a wide range of possibilities for the future use of plant-derived vesicles in health and therapeutics. Not only can they be potentially used for inflammatory diseases such as IBD and colitis, but plant-derived vesicles may have other promising uses in serious and life-threatening diseases, including cancer. The current research that exists for EVs from ginger and grapefruit should be expanded to other edible plant food sources.

### 1.4. Plant Vesicles: Critical Thinking and Challenges in the Research Field

While plant-derived extracellular vesicles have been shown to have numerous benefits and potential future uses, there are still many reasons why these EVs are not yet being used on a large-scale basis, including challenges and knowledge gaps that have arisen amongst researchers in this field. In this section, we will discuss and elaborate on some of these challenges.

Firstly, one of the main problems that researchers in this field face is the issue of protocol standardization. Many techniques are currently used to isolate EVs, including ultracentrifugation, density gradients, and, more recently, size exclusion chromatography (SEC) [39]. While each method has its benefits, there are also some drawbacks that are associated with these protocols. For example, while ultracentrifugation is a common and cost-effective procedure, there is a risk of disrupting EVs beyond a certain speed and force, which must be considered [40]. Additionally, compared to other techniques, ultracentrifugation is a time-consuming procedure, may result in a lower yield of vesicles, and it also raises the problem of co-contamination with unwanted proteins [41]. There have reportedly been inconsistencies in the results obtained by researchers using ultracentrifugation for EV research, and these may also be affected by centrifugation speed, force, and the type of rotor [41,42]. Nevertheless, ultracentrifugation is one of the gold standard procedures used for plant vesicle isolation, and when combined with other methods, including sucrose gradients and SEC, some of the cons associated with ultracentrifugation are reduced. Sucrose density gradients are also being increasingly used to isolate plant vesicles [14,18,20,21,22], which allow vesicles to be separated and collected based on their density [41], resulting in the collection of purer samples. However, sucrose density gradients can be extremely time-consuming, and may result in a lower yield of vesicles, although with a higher purity [43].

Further characterization of EVs to study their role in physiological and/or pathological processes is one of the major challenges in the EV field. Since proteins are the building blocks that determine, to a large extent, the function of EVs in biological systems, proteomics profiling can advance our understanding of EVs in biological settings. This was a major challenge until recently due to the nanoscale size of the EVs; however, because of recent advances, it is now possible to quantify EV proteins from small numbers of EVs. These advances involve (besides innovated flow cytometry) newly emerging technologies for EV protein quantification, such as optical, non-optical, microfluidic, and single vesicle detection methods [44]. However, most of these detection methods rely on well-known markers of EVs. In mammalian cells, CD63, CD81, and CD9 are among the most common markers, while bacterial outer membrane vesicles can be identified by markers such as outer membrane protein A (OmpA) and lipoteichoic acid (LTA) [45]. Yet, to our knowledge, there is significantly less information on target marker proteins when it comes to plant vesicles [28,38]. One study has investigated the presence of surface proteins on citrus-derived vesicles and was able to successfully identify some common marker proteins, including patellin-3-like proteins and clathrin heavy chain proteins [28,46]. However, other studies have found contradicting results. Due to the inconsistencies in findings on PDEV protein composition and the limited information on plant EVs, more research is warranted.

In summary, there is no doubt that the already-existing technologies originally designed for mammalian EVs can also be applied for the isolation and characterization of PDEVs. In addition, PDEV research has yielded some exciting findings which may have relevant applications in the future. However, it is important to remember that while the current results are promising, there are still many knowledge gaps that need to be filled, which will give more insight into where plant EVs may fit in a clinical setting. Nevertheless, it is evident that research on plant EVs is definitely worthy of further investigation, and these challenges are pushing researchers in the right direction.

## 2. Conclusions and Future Outlooks

In the last decade, there has been an exponential increase in the research being conducted on plant-derived extracellular vesicles [15]. Previous studies have shown that PDEVs are diverse, highly complex, and offer a range of interesting possible future applications. Nevertheless, these nano-sized vesicles, which have been isolated from a variety of plant sources, may offer a multitude of possible applications in health, disease, and drug delivery. Although some challenges with respect to characterization and identification must be overcome, the already-existing information on plant vesicles is so promising that the continuation of research in this field is worth expanding on in the future.

## Figures and Tables

**Table 1:** Results from preliminary research on plant-derived EVs and their effects in vitro and in vivo.

Plant Source	In Vitro Experimental Findings	In Vivo Experimental Findings	Reference
Ginger	Taken up by cancer cells, intestinal epithelial cells, and macrophages	Targeted delivery of doxorubicin to tumors Enhanced chemotherapeutic inhibition of tumors	[25]
		Reduced DSS-induced colitis and pro-inflammatory cytokines (TNF-α, IL-6, IL-1β) Increased anti-inflammatory cytokines (IL-10, IL-22)	[24]
		Protected against alcohol-induced liver damage	[34]
Grapefruit	Taken up by various cell lines without inducing cytotoxic responses	Delivered chemotherapeutic agents, short RNAs, and proteins to various cells Did not pass the placental barrier of pregnant mice	[26]
		Taken up by intestinal macrophages Reduced DSS-induced mouse colitis	[26]
Grape	-	Taken up by intestinal stem cells and promoted intestinal stem cell proliferation Protected against DSS-induced colitis Promoted the expression of the anti-inflammatory cytokine IL-10	[14,21]
Citrus lemon	Inhibited cancer cell proliferation	Suppressed tumor growth	[22]
Broccoli		Protected against DSS-induced colitis	[35]

## Data Availability

Not applicable.
